# Evaluation of the inter-examiner reliability of myofascial trigger point identification in dogs

**DOI:** 10.3389/fvets.2026.1782274

**Published:** 2026-02-27

**Authors:** Bryce Talsma Roberts, Christina Montalbano, Felix Michael Duerr, Karolynn Mireya Ellis, Lindsay Hochman Elam

**Affiliations:** 1 Department of Clinical Sciences, Colorado State University, Fort Collins, CO, United States; 2 Department of Comparative, Diagnostic, and Population Medicine, University of Florida, Gainesville, FL, United States; 3 Department of Clinical Sciences, College of Veterinary Medicine, Cornell University, Ithaca, NY, United States

**Keywords:** dog, MTrP, myofascial, reliability, trigger point

## Abstract

**Introduction:**

Myofascial trigger points (MTrPs) are a source of chronic pain in humans, but their diagnosis relies on subjective manual palpation. MTrP distribution has been described in dogs, but reliability between examiners remains unknown. This prospective, blinded, clinical investigation aimed to determine the inter-examiner reliability of MTrP identification in the hindlimb musculature of dogs and to describe their distribution.

**Methods:**

Twenty-four geriatric and retired sled dogs were assessed for MTrPs in four hindlimb muscle groups (gluteals, cranial thigh, hamstrings, and medial thigh) by two veterinarians in random order. MTrP identification was based on previous methodology defining an MTrP as a distinct, hyperactive point or nodule eliciting a pain response, known as a jump sign. Examiners were blinded to each other's findings by marking MTrPs with invisible UV ink pens. Agreement on MTrP presence or absence in a muscle group, distance between examiners' points, and the number of MTrPs per muscle group were recorded. Inter-examiner reliability was assessed using Cohen's kappa.

**Results:**

In total, 380 MTrPs were identified by the two examiners across the 188 muscle groups assessed. Examiners demonstrated 81.4% agreement on MTrP presence or absence in a muscle group with a Cohen's kappa of 0.608 (95% CI: 0.491–0.724), indicating moderate to substantial inter-examiner reliability. However, the mean distance between examiners' markings for individual MTrPs was 10.6 ± 5.1 mm, suggesting a potential substantial lack of precision. The cranial thigh group contained the most MTrPs. Additionally, the more experienced examiner identified a greater number of MTrPs overall (*p* = 0.028).

**Discussion:**

This study provides the first assessment of inter-examiner reliability for MTrP identification in dogs. While examiners agreed on the general presence or absence of myofascial sensitivity within a muscle group, precise localization was unreliable. This lack of precision may be influenced by a number of factors, namely skin movement and the subjective nature of the assessment itself. This study highlights the need for further research and objective diagnostic tools for reliable, targeted MTrP identification.

## 1 Introduction

Myofascial pain syndrome (MPS) is described as an often chronic, musculoskeletal pain condition in which there is regional pain within a muscle, fascia, or the surrounding soft tissue. In humans, myofascial pain is commonly recognized as a key component of chronic pain (1) and is primarily characterized by the presence of discrete myofascial trigger points (MTrPs) and fascial restrictions that are sensitive to stimuli, causing localized and/or referred pain (2, 3). MTrPs are defined as nodules within taut bands of skeletal muscle that can be spontaneously painful (i.e. active), or only painful on direct compression (i.e. latent) (4). The pathophysiology of MPS appears complex and incompletely understood, but several theories have emerged to propose mechanisms for the development of MTrPs. One of the most accepted theories suggests overload to the muscle fibers caused by overuse from repetitive or prolonged activity. Consequently, there is tissue hypoxemia and ischemia, leading to calcium pump dysfunction and an “energy crisis” within the muscle fiber, resulting in sustained contraction of the fiber and taut band formation (4). The clinical manifestations of MTrPs in dogs, extrapolated largely from human medicine, include pain and motor abnormalities (e.g. reduced range of motion, muscle weakness) (5). Palpation of a MTrP can elicit a characteristic pain response, such as vocalization, withdrawal, or a local twitch response in dogs (6, 7).

Despite the clinical relevance of MPS to both pain management and physical rehabilitation, the concept of MPS and MTrPs remains a poorly understood problem in human and veterinary medicine alike. In humans, pain localization often relies on verbal cues, whereas the veterinary assessment often relies on more subtle behavioral cues to localize pain, further complicating precise localization. The clinical distribution of MTrPs have been described in dogs and horses (6, 8–10). However, there remains a dearth of scientific literature concerning the reliability of MTrP identification between observers to aid in understanding its diagnosis and possible treatment of this condition in animals.

Crucially, the diagnosis of MTrPs relies primarily on manual palpation with no widely accepted diagnostic test to objectively identify these points (11). This inherently subjective assessment introduces a major limitation, particularly in veterinary medicine where pain perception and expression are influenced by both physiological and behavioral factors (12). In humans, inter-examiner reliability of MTrP identification is variable and often unreliable (13), although the use of invisible ink pens has been shown to be an effective research tool (14, 15). Inter-examiner reliability of MTrP identification has not yet been investigated in dogs, presenting both a diagnostic challenge and an opportunity to improve understanding and recognition.

The primary aim of this prospective, blinded, clinical investigation was to identify the inter-examiner reliability of MTrP identification in dogs. A secondary aim of this study was to describe the frequency of MTrPs in four different hindlimb muscle groups of the dog.

## 2 Materials and methods

### Study population

The study protocol and procedures were approved by the Cornell University Institutional Animal Care and Use Committee (IACUC #2018-0022). Twenty-six geriatric and retired Alaskan sled dogs were examined for enrollment. All dogs were retired approximately 6 years prior to data collection, housed in the same facility, fed one of two commercial diets to maintain an ideal body condition (16), and allowed similar daily activities. While the nutrient profile of the diets differed, all dogs had been consistently receiving this diet prior to and throughout data collection. All medications were kept consistent prior to and throughout data collection as well. Activities included 30 min of free roaming twice daily in one of three fenced yard spaces covering one acre total, and a few other brief social interactions throughout the day. Dogs were excluded if they were behaviorally intolerant of physical and orthopedic examinations or were unable to stand for the duration of the examinations (approximately 10 min). The medical records were reviewed, including full body computed tomography (CT) scans performed within 2 years with all remarkable musculoskeletal findings documented.

At least 48, but no more than 72 h prior to MTrP evaluations, full physical and orthopedic exams were performed by a single examiner (BR). Any musculoskeletal abnormalities were recorded in addition to the dog's weight, body condition score (BCS), muscle condition score (MCS), and withers height (17, 18). At the conclusion of the examination, the hindlimb fur of each dog from the iliac crest through the distal aspect of the tibial tuberosity was clipped on both sides.

### Myofascial trigger point identification

**Figure 1:**
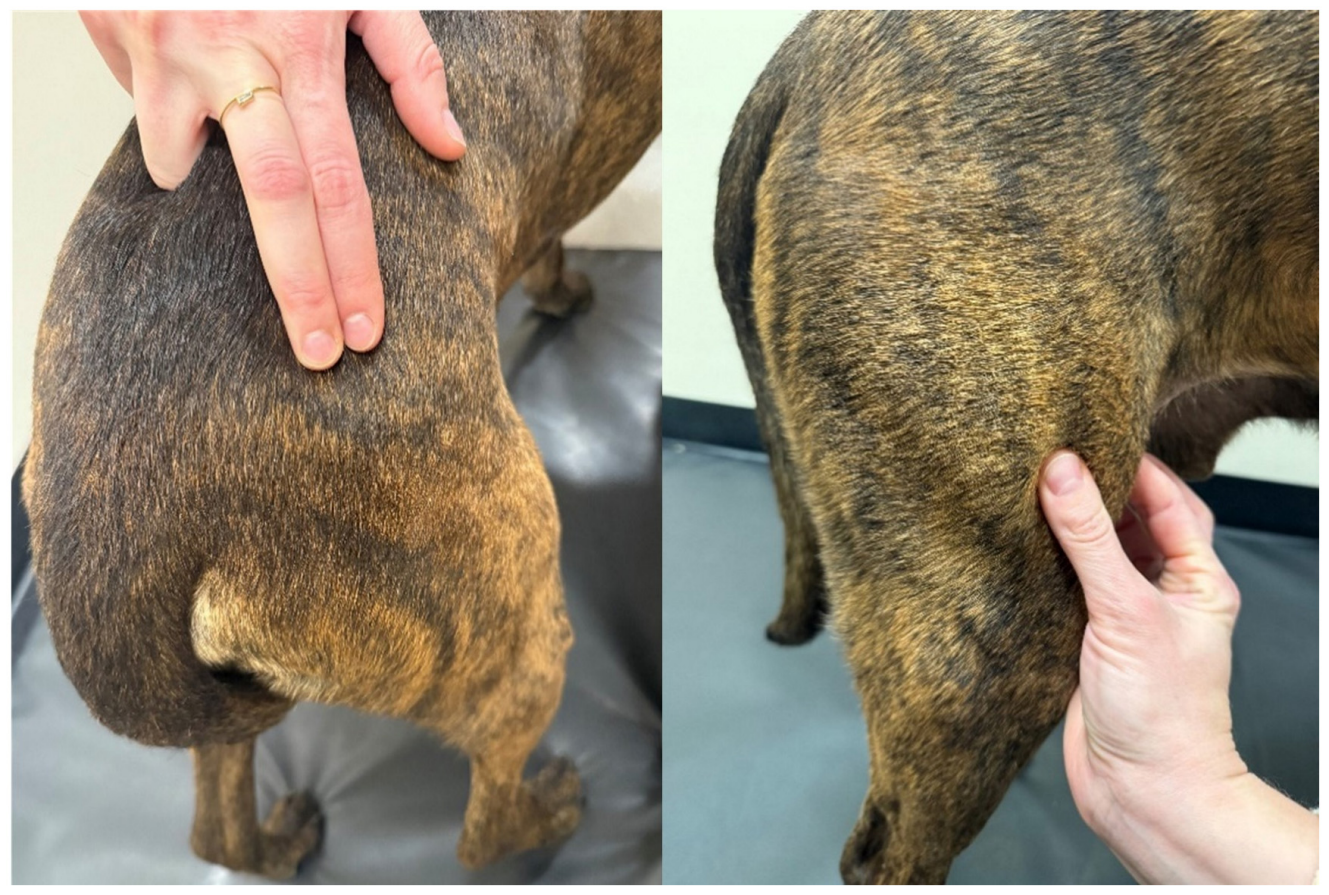
Demonstration of the flat **(left)** and pincher **(right)** palpation techniques.

Examiners consisted of one ACVSMR diplomate (CM) with 8 years of clinical experience in myofascial examinations and one ACVSMR resident (BR) with 3 years of experience. Each examiner was given a written description of the palpation technique and landmarks for each muscle with the flat and pincer techniques used as previously described (Table 1; Figure 1) (7, 19). Similar to the methodology described by Formenton et al. a MTrP was defined as a distinct hyperactive point or nodule that elicits a contractile response suggestive of pain (jump sign, Supplementary Video 1). Pain was defined as vocalization, turning the head toward or looking at the target area or examiner, flexing the spine, or exhibiting an escape behavior during trigger point palpation.

**Table 1:** Description of the muscle landmarks for muscle palpation, palpation technique, and the muscle group characterization.

**Muscle group**	**Muscle**	**Palpation landmarks**	**Technique**
Gluteals	Superficial gluteal	From the sacrum and first caudal vertebrae traveling distally until its tendinous insertion on the greater and third trochanters	Flat
	Middle gluteal	From the iliac crest and tuber sacrale traveling caudally and distally over the gluteal surface of the ilium until it is covered by the superficial gluteal muscle caudally and before its insertion on the greater trochanter	Flat
Cranial thigh	Sartorius	From the origin on the ilium at the iliac crest, ventral iliac spine, and tuber coxae distally to the insertions on the medial femoral fascia just proximal to the patella and aponeurosis and crural fascia on the cranial and proximal part of the tibia	Pincer
	Quadriceps	From the origin on the body of the ilium and proximal femur distally through its insertion on the patella	Pincer
Hamstrings	Semitendinosus	Beginning proximally at the ischiatic tuberosity until the insertion on the medial aspect of the tibia at the aponeurosis of the gracilis muscle. The tendinous continuation to the calcaneus will not be palpated	Pincer
	Semimembranosus	Beginning proximally at the ischiatic tuberosity until the insertion at the medial lip of the distal end of the femur and medial femoral condyle down through the medial condyle of the tibia	Pincer
Medial thigh	Adductor	Beginning proximally at the level of the pelvic symphysis distally and laterally until it dives under the pectineus and caudal part of the sartorius	Flat
	Pectineus	Beginning proximally at the iliopubic eminence palpated distally until the myotendinous junction where it dives under the caudal part of sartorius	Pincer
	Gracilis	Beginning proximally at the level of the pelvic symphysis region palpated distally until its aponeurosis on the medial aspect of the stifle. The tendinous continuation to the calcaneus will not be palpated	Pincer

### Myofascial trigger point evaluation

For the myofascial trigger point evaluation, the patient was placed so the dog was standing symmetrically with the hindfeet under the hips and forefeet under the shoulders. The nose was maintained at the level of the wings of the ilium. Foot placement was marked with tape at the lateral aspect of the fifth digits to ensure consistent patient position between examiners. An unblinded individual not involved in palpation (KE) was present for gentle restraint and to ensure patients remained as stationary as possible. Palpation began with the gluteal muscle group (superficial and middle gluteal muscles), followed by the cranial thigh (sartorius and quadriceps muscles), then hamstrings (semitendinosus and semimembranosus muscles), and ended with the medial thigh (gracilis, adductor, and pectineus muscles). Evaluations always started on the right side for both examiners to ensure the same leg had been palpated if a dog became behaviorally intolerant to continued palpation.

**Figure 2:**
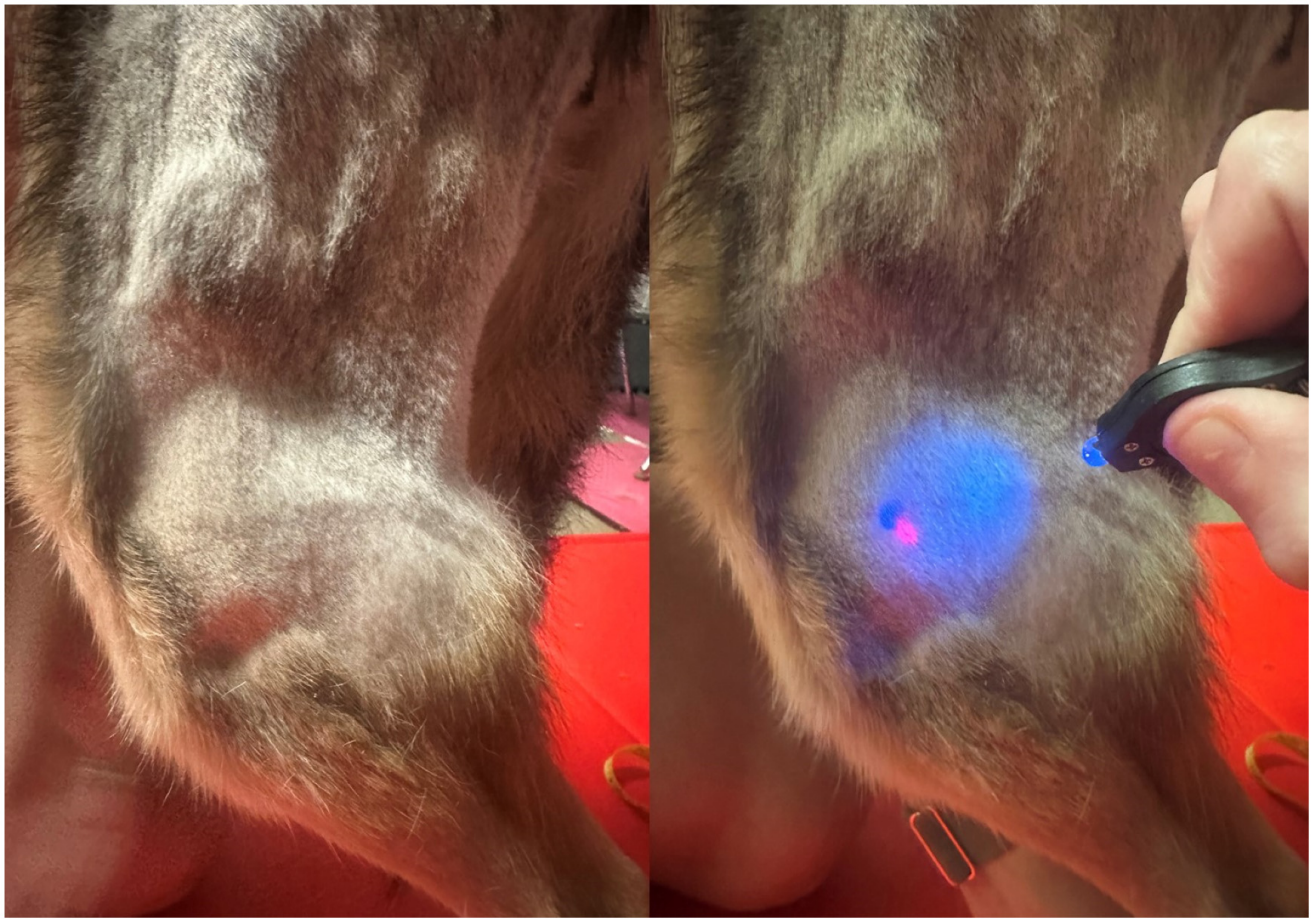
The shaved left stifle joint showing the marking of the fibular head using two different colored UV ink markers. The left image demonstrates visualization under fluorescent lighting, and the right image highlights the points with UV light.

Invisible ink UV markers of varying colors with an approximately 2 mm diameter tip (DirectGlow, Dayton, OH, United States) were used to mark the skin at identified MTrPs. These marks dried instantly, were not visible under fluorescent lights, but were clearly visible when illuminated with UV light (Figure 2), allowing for blinding between examiners. Each examiner used the same marker throughout the procedure. Each examiner additionally marked the most proximal, palpable aspect of the fibular head as a defined, fixed, bony landmark.

The order for examiner palpation was randomized for each dog using a coin flip. The first examiner palpated each muscle, as described, perpendicular to the direction of the muscle fibers. If a MTrP was identified, digital pressure was removed from the muscle and the spot was marked with a small dot approximating the 2 mm diameter of the marker tip. Palpation continued for the remainder of the muscle with further MTrPs marked in a similar manner. The entire muscle was palpated only once to avoid repeated compression and potential release of the MTrP (20). After palpating each muscle group, the examiner recorded the number of MTrPs identified. Following the first examiner's evaluation, the second examiner, who had been waiting in a separate room, immediately followed in the same fashion using a different colored UV ink marker.

### Measurements

**Figure 3:**
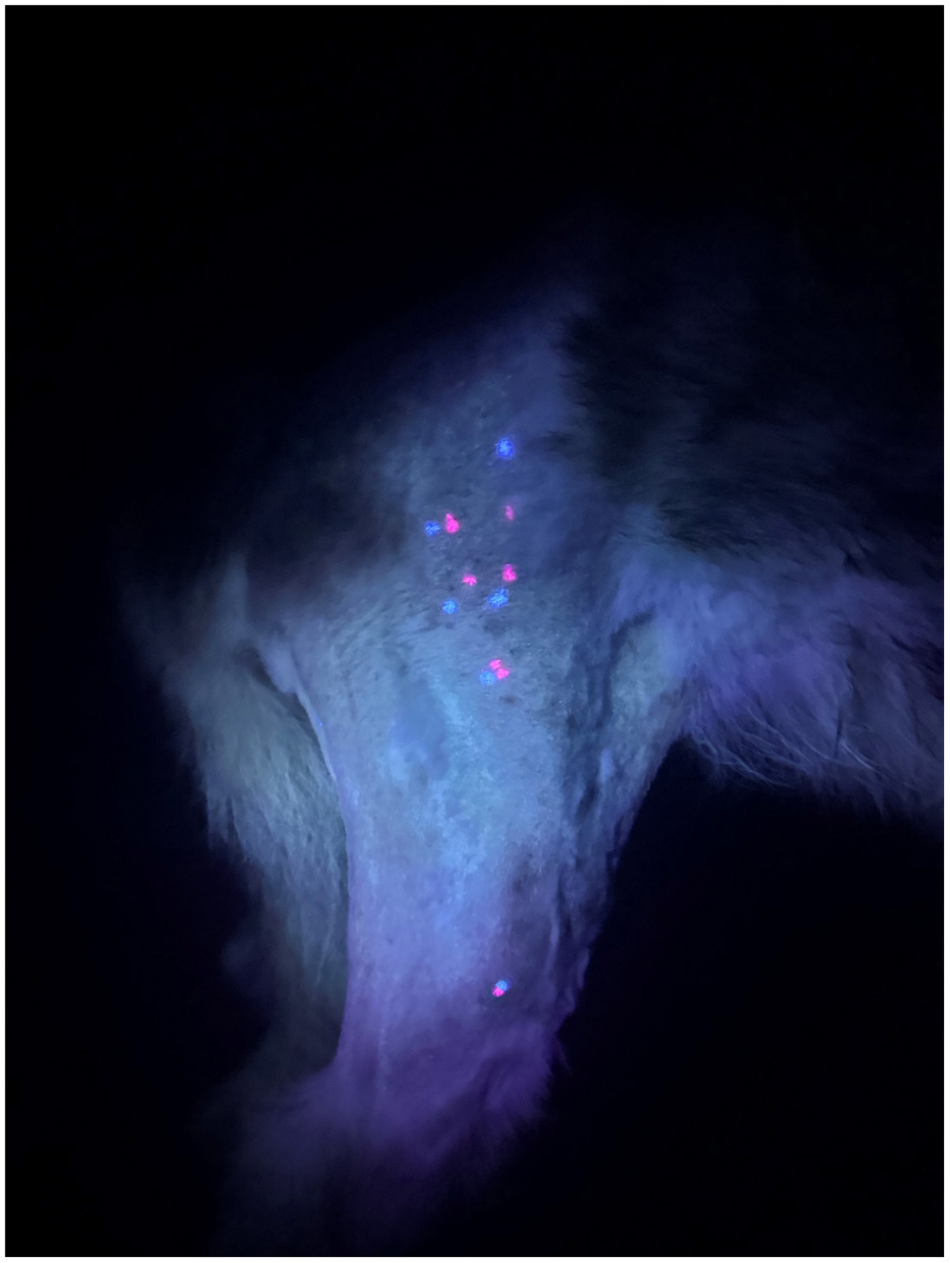
Example of the illumination of MTrPs in the cranial thigh (proximally) and markings on the fibular head (distally) following palpation by both examiners.

At the conclusion of the examinations, the UV light was used to illuminate the marked points on the muscle groups and fibular head (Figure 3). The number of MTrPs in each muscle group as well as the total for each examiner were counted, and the distance between identified MTrPs was measured. If there was no regional MTrP within 20 mm or the other evaluator did not mark a MTrP for that muscle group, no distance measurement was recorded and the point was classified as “no comparison,” but the MTrP was still counted for that examiner. The distance between each examiner's fibular head landmark point was also measured from the center of one mark to the other in millimeters (mm).

### Statistical analyses

Sample size calculation estimated a MTrP prevalence of 85% in dogs (8), and a minimum kappa of 0.6 (21). The sample size needed to achieve a level of confidence of 95% and precision of 0.2 was 124 muscle groups.

All statistical analyses were conducted using R software (22) including the following packages: *tidyverse, readxl, openxlsx, lme4, magrittr, broom*, and *psych*. Muscles were grouped for analysis as gluteals, cranial thigh, hamstrings, and medial thigh (Table 1). Summary statistics were calculated for all numerical response variables overall, by leg side, by muscle group, and by leg side and muscle group. The numerical response variables included the count of MTrPs identified by each examiner and the distance between points identified by each examiner. Summary statistics were calculated for the distance between individual MTrPs in each muscle group.

The proportion of agreement between the examiners was estimated overall, relative to each leg side, relative to each muscle group, and relative to both leg side and each muscle group. A positive agreement was defined in two ways:

1. Both examiners marked at least one MTrP in the muscle group.
2. Both examiners did not mark any MTrPs in a muscle group.

Cohen's kappa (95% confidence interval) was estimated to assess the inter-examiner reliability for the presence/absence identification of MTrPs. A chi-squared test of association and Fisher's exact test was estimated to assess if there was an association between the examiner and the identification of MTrPs. Bonferonni multiple adjustment was then applied when testing relative to each leg and when testing relative to each muscle.

A general linear mixed model (Poisson) was fitted to determine if there were any associations for the number of points identified by each examiner. The leg side, muscle, and examiner were considered fixed effects, and dog ID a random effect. All two-way interactions between the fixed effects were also considered.

## 3 Results

Of the 26 dogs evaluated for enrollment, 24 dogs were included for data analysis. The population comprised of five intact males, five neutered males, and 14 spayed females. Ages ranged from 13 to 16 years (median = 14 years), withers height from 51 to 62 cm (median = 57 cm), and weight ranged from 17.4 to 30.3 kg (median = 23.0 kg). BCS ranged from three to five out of nine (median = 5) and MCS ranged from two to three out of three (median = 2). Relevant clinical diagnoses and medications are summarized in Table 2 for each dog. One dog was excluded during initial assessments due to behavioral intolerance of the standing assessment, and one dog was excluded on the day of palpation exams due to an acute onset of non-weight bearing hindlimb lameness attributed to cranial cruciate ligament disease. One additional dog became intolerant of palpation during examination, and the left side was not completed by both examiners, so a total of 47 limbs were included for analysis. In total, 423 muscles within 188 muscle groups were assessed.

**Table 2:** Summary of remarkable patient exam, CT findings, and clinical history.

**Patient #**	**Remarkable exam findings**	**Clinical diagnoses**	**Medications**
1	Left shoulder pain, thoracolumbar pain, left tarsometatarsal thickening	Mild bilateral tarsal osteoarthritis, lumbar intervertebral disc disease	None
2	Amputation of left front digit 2, lumbosacral pain	Historic osteosarcoma of the left front digit 2, hypothyroid	Levothyroxine
3	Resistant to hip extension, reduced spinal mobility, flattened digits	None	None
4	Left flexor carpi ulnaris thickening with pain on palpation and carpal flexion, hypomobile spine	Lumbar intervertebral disc disease	None
5	Lumbosacral pain, delayed conscious proprioception in hindlimbs, right shoulder pain	Lumbosacral disease	None
6	Right front limb lame, right shoulder pain, lumbosacral pain	Transitional thoracolumbar vertebrae	None
7	Mild thoracolumbar pain	None	None
8	Multifocal spinal pain	None	None
9	Lumbosacral pain	None	None
10	Thoracolumbar and lumbosacral pain, reduced spinal mobility, tarsometatarsal thickening bilaterally	None	None
11	Bilateral tarsometatarsal thickening (left worse), reduced spinal mobility, resistant to hip extension, mild lumbar spinal pain	Lumbar intervertebral disc disease	None
12	Unremarkable apart from very mild thoracolumbar spinal discomfort	None	None
13	Unremarkable exam	None	None
14	Resistance to hip extension, possible mild lumbosacral discomfort, otherwise unremarkable	Transitional thoracolumbar and lumbosacral vertebrae	None
15	Resistance to hip extension, otherwise unremarkable	Transitional vertebrae, historically removed soft tissue sarcoma of right front digit	None
16	Overgrown nails, mild lumbosacral and thoracolumbar pain	None	None
17	Mild spinal discomfort	Lumbar spondylosis	None
18	Left hip resistance to extension, left shoulder discomfort, lumbosacral discomfort	Multifocal intervertebral disc disease, lumbosacral disc extrusion	None
19	Lumbosacral pain, left carpal thickening, no pain or reduced range of motion	Left carpal osteoarthritis	None
20	Tarsometatarsal thickening, spinal hypomobility, moderate lumbosacral pain	Bilateral tarsal osteoarthritis, cervical intervertebral disc disease	None
21	Thickening left medial tarsus vs. mass, reduced tarsal flexion with pain bilaterally, left stifle medial buttress without instability, spinal pain	Historic immune-mediated polyarthritis now in remission, left stifle most affected	None
22	Lumbosacral pain	Multifocal intervertebral disc disease with lumbosacral subluxation, chronic left front lameness	Carprofen, gabapentin
23	Right front limb significant atrophy, mild shoulder pain, right cervical paraspinal atrophy, resistance to right front limb manipulation in general	Right stifle osteoarthritis, historic vestibular disease and right front limb lameness, hyperadrenocorticism	Trilostane
24	Tarsometatarsal thickening, spinal hypomobility, mild lumbosacral pain	Cervical and lumbosacral intervertebral disc disease	Amitryptiline (anxiety), oclacitinib

### Trends and Associations for MTrP Identification

**Figure 4:**
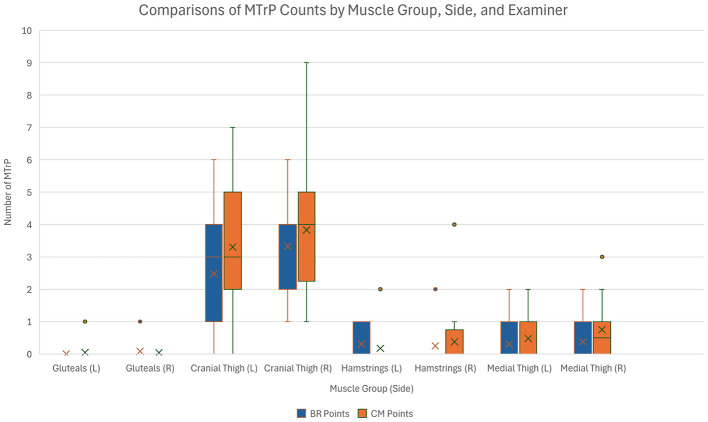
A box and whisker plot demonstrating the distribution of MTrP counts for each examiner, sorted by muscle group and side.

All dogs had at least one MTrP identified by both examiners, but there was moderate variability in the number of MTrPs identified among individual dogs (Range = 5–41, SD = 0.420). Differences were also observed in this population when considering the number of MTrPs identified by each examiner, by leg side, and by muscle group which are summarized in Figure 4. A total of 380 MTrPs were identified between the two examiners (BR = 168, CM = 212) with the more experienced examiner identifying more MTrPs (*p* = 0.028). There were more MTrPs identified on the right side relative to the left (*p* = 0.022), and there was evidence of more MTrPs being identified in each of the other muscle groups when compared with the gluteal muscles (*p* < 0.001 for all). The cranial thigh contained the most MTrPs per leg (3.24 ± 1.89), followed by the medial thigh (0.48 ± 0.79), hamstrings (0.27 ± 0.61), and gluteals (0.04 ± 0.20).

While there were factors that influenced the number of MTrPs, whether or not a MTrP was identified was not associated with the examiner (*p* = 0.52), the leg side (*p* > 0.5 for both left and right), or the muscle group (*p* > 0.2 for all muscle groups).

### Agreement within muscle groups

All dogs had at least one MTrP identified in at least one muscle group by both examiners. A summary of MTrP identification by muscle group and proportions of agreement based on the identification of a presence or absence of MTrPs is provided in Table 3. Overall, examiners agreed on the presence or absence of MTrPs in a given muscle group 81.4% of the time. Of this agreement, 64.1% of the time there was an absence of MTrPs, and 35.9% there was a presence of MTrPs. When considering agreement at the level of the muscle group, differences were observed. The gluteal muscle group had the highest agreement at 95.7%, but nearly always agreement was based on the lack of MTrPs. Agreement in the cranial thigh muscle group was 87.2% with MTrP being present in all instances of agreement. The hamstrings and medial thigh muscle groups had similar overall agreement on presence or absence of MTrPs at 72.3 and 70.2%, respectively, but agreement and MTrP presence was higher in the medial thigh group at 27.3% compared to 11.8% in the hamstrings group. The Cohen's kappa coefficient for inter-examiner reliability overall was 0.608 (95% CI: 0.491–0.724), indicating moderate to substantial agreement between examiners overall.

**Table 3:** Summary of MTrP identification in each muscle group with proportions of agreement between examiners on the presence or absence of MTrPs.

**Muscle group**	**MTrPs identified per observer**	**Total number of MTrPs identified**	**Overall agreement (%)**	**Presence of MTrP with agreement**	**Absence of MTrP with agreement**
Gluteals	BR: 2 CM: 2	4	95.7	2.2%	97.8%
Cranial thigh	BR: 137 CM: 168	293	87.2	100%	0.0%
Hamstrings	BR: 13 CM: 13	26	72.3	11.8%	88.2%
Medial thigh	BR: 16 CM: 29	45	70.2	27.3%	72.7%

The mean distance between fibular head points averaged 10.6 ± 7.6 mm overall with an average of 12.7 ± 8.9 mm on the left and 8.7 ± 5.5 mm on the right. Considering when at least one examiner identified a MTrP in a muscle group, 251 comparisons were analyzed. Of these 251 comparisons, 83 measurements were within 20 mm. The mean distance between these MTrPs was 10.6 ± 5.1 mm. On the left side, the mean distance between measured MTrPs was 10.2 ± 5.6 mm, and on the right side 10.9 ± 4.7 mm.

## Discussion

This study investigated the inter-examiner reliability of MTrP identification in the hindlimb muscles of dogs and characterized the distribution of MTrPs among muscle groups. The findings suggest moderate to substantial agreement on the presence or absence of MTrPs within a given muscle group of the hindlimbs, but there was a lack of precision in identifying the same anatomical point of the MTrP.

Overall, the examiners could consistently recognize the general presence of myofascial sensitivity in a muscle group, but agreement on exact location of a given MTrP was unreliable. Two possible explanations for these findings may be inaccuracies in examiners identifying the MTrPs (i.e. differences in palpatory findings) or erroneous methods used to mark them (i.e. differences with marking MTrPs accurately). Supporting the latter, the distance between examiners' identification of the fibular head—an objectively fixed and immovable landmark—was also variable with an averaging distance between marked points of more than 10 mm overall. This suggests factors such as skin displacement or postural changes in the dog likely contributed to measurement discrepancies. Given the ample subcutaneous tissue in dogs, movement of skin during palpation and during even subtle changes in posture may have deviated the marking from its true origin. Further complicating this issue is the subjective nature of interpreting pain responses to identify MTrPs. In human studies, patients can verbally describe local or referred pain, pain intensity, and pain recurrence, providing clearer information on MTrP presence and location (15). In contrast, identification in this study relied on the interpretation of behavioral cues in dogs that may have indicated pain. Additionally, because MTrPs are known to cause referred pain, determining the exact location, rather than just its presence or absence, is inherently more challenging (23).

Another potential contributor to the low agreement on precise location of MTrPs may be the difference in clinical experience between examiners. The more experienced examiner (CM) identified more MTrPs than the less experienced examiner (BR). These results are similar to a study assessing MTrP identification in people where examiner experience was shown to influence inter-examiner reliability (15). While both examiners in this study had experience in, and regularly perform, myofascial examinations, clinical experience does appear to influence tactile sensitivity (24). Additionally, clinicians were not told how much pressure to apply during their palpations. The pressure applied during palpation may have differed between clinicians and impacted the appreciation of MTrPs. As such, improved training and standardization in the clinical assessment of MTrPs may help enhance diagnostic consistency. However, given the lack of a true gold standard for identifying MTrPs, the actual number of MTrPs present in the study population is unknown. Future studies may consider assessing reliability using more examiners that consist of both similar and varying levels of experience to determine if the findings in this study can be applied more broadly. Further, this study did not evaluate the intra-examiner reliability of MTrPs. A better understanding of intra-examiner reliability may further elucidate potential factors in MTrP identification variability. To date, no studies have evaluated the intra-examiner reliability of MTrPs in dogs. In humans, intra-examiner reliability was found to be similar to inter-examiner reliability for evaluation of the lower leg muscles (25). However, a better understanding of the intra-examiner reliability in dogs is crucial considering assessment, intervention, and follow-up care are often made by the same clinician.

To date, no imaging modality has proven to consistently and reliably identify MTrPs. Conventional grayscale (B-mode) ultrasound is currently considered the most promising imaging modality for MTrP assessment given the emerging evidence on efficacy, cost, and ease of use (26). Ultrasound may identify MTrPs as regions with altered echotexture, but studies have yet to consistently associate ultrasonographic and palpation findings (27). Vibration or shear-wave elastography with ultrasound has been used to measure the stiffness of tissue in presumed MTrPs (28). The vibration propagates shear-waves in the tissue of interest, and the pattern is displayed as a color variance image using Doppler techniques (29). However, a recent systematic review in human medicine found inconsistencies in the elastography imaging protocols and varying MTrP imaging characteristics (26, 30). Across studies, the sensitivity and specificity of ultrasonographic techniques varied widely, suggesting standardization of imaging protocols and higher quality methodological studies are needed to better understand the potential utility of ultrasound in diagnosing MTrPs (26). Infrared thermal imaging has been proposed as a non-invasive method for visualizing regional temperature patterns that may be suggestive of altered microcirculation, but this method currently lacks strong clinical validation and can be influenced by external factors (3). Other diagnostic imaging tools have also been explored such as magnetic resonance imaging (MRI), electromyography (EMG), and microdialysis, but these are more costly and/or invasive, limiting their clinical utility (3, 31–34).

While advances in diagnostic modalities will refine our ability to identify and characterize MTrPs, understanding their anatomical distribution provides some insight into their functional relevance and potential pathologic etiologies. In this study, the cranial thigh region yielded the highest number of MTrPs. This aligns with previous findings in working dogs and those with osteoarthritis (6, 8) and may reflect the functional role and biomechanical loading of these muscles. The cranial thigh muscles are critical to weight-bearing and stabilization, especially in postural control, and their predominance in MTrP distribution may relate to fiber-type composition, chronic loading, or neuromuscular tension patterns, as has been theorized in human studies (1, 4). According to Henneman's size principle, motor units are recruited in order from smallest to largest depending on the intensity of the force being applied; smaller, type I muscle fibers will be recruited first and de-recruited last in static muscle exertions such as postural control (35). In one theory on MTrP formation, these overworked fibers are metabolically overloaded from the sustained activation and more susceptible to MTrP formation. This is in comparison to larger muscle fibers that do not work as hard and spend less time activated (4). This has been supported in human literature where computer workers were found to have a higher frequency of MTrPs in their trapezius muscles, a key postural muscle in humans (36). While different muscles were evaluated in this study, a similar theory may support why the cranial thigh muscles had a higher number of MTrPs identified by both evaluators.

The patient population utilized in this study may have also impacted results. The dogs were relatively homogenous in age, breed, and lifestyle, but there was variability in their diagnosed musculoskeletal conditions. In human medicine, lower levels of physical activity have been associated with a higher prevalence of MTrPs (2, 3). Poor muscling or chronic changes in soft tissue compliance may have affected the ability to detect MTrPs (37), potentially reducing the inter-rater agreement, but it remains unclear how muscle condition or prior work history influences MTrP prevalence and distribution in dogs.

This investigation also revealed that more MTrPs were identified on the right hindlimb compared to the left. While the study design controlled for palpation order for examiners, the right side of the dog was always palpated first. Palpation fatigue may have contributed to fewer MTrPs identified on the left as this side was palpated last for both examiners. Hand dominance or body position may have played a role in MTrP identification, but both examiners utilized both hands at different times depending on the muscle assessed. Apart from examiner factors, the patient population may have also influenced the right-sided MTrP distribution. Formenton et al. also found more MTrPs on the right side of working dogs, attributed to their working conditions. This sled dog population was now retired and much less active, so work less likely explains the side differential, but the concurrent musculoskeletal conditions may have contributed to the laterality as MTrPs are known to develop in response to repetitive strain, postural imbalance, or spinal pathology (37). The role of different nutrient profiles in the diet and concurrent medications were not investigated in this study, but these factors were held consistent prior to data collection with no lapse in routine medical management for underlying conditions. Interestingly, the dog with the highest number of MTrPs in this study was concurrently being treated for hypothyroidism. In humans, hypothyroidism has been linked with a predisposition for MTrP formation (3). Future studies may further evaluate the role these factors play in MTrP identification by randomizing the side of first palpation and evaluating dogs with single, unilateral musculoskeletal conditions. While these factors may have influenced the number of MTrPs, it was unlikely to impact the inter-examiner reliability during this single point of data collection.

This study did not assess the clinical response to treatment of identified MTrPs. The importance of precise and reliable MTrP identification depends on the type of treatment utilized with precise identification being crucial for targeted therapies such as acupuncture. Currently, no studies exist to evaluate the effectiveness of any MTrP therapies in dogs, so reported therapies are strictly anecdotal (2, 7, 19). Both dry and wet needling have been suggested as a targeted treatment for MTrPs in human and veterinary medicine with wet needling using a local anesthetic such as lidocaine (19, 38). Ischemic compression is another targeted therapy in which digital compression of the MTrP for 60–90 s with increasing pressure has been used as a treatment for MTrPs in various muscles (38–41). When considering treatment of specific MTrPs, reliable localization of MTrPs would be imperative to optimize potential success of these more precise and focal therapies. However, for other more generalized treatment modalities that have been described such as extracorporeal shockwave therapy, stretching, and manipulative therapies (2, 7, 42), the moderate to strong agreement between examiners of affected musculature demonstrated in this study could be adequate to guide therapy.

Several additional limitations of this study should be acknowledged. While this patient population was relatively homogenous, extrapolation of these results to a more general population of dogs and athletic dogs should be exercised with caution. Sampling a population of client-owned dogs in this study was not feasible given the necessity to clip a large portion of the hindlimbs. The interpretation of the jump sign also remains subjective. Additional criteria such as the palpation of a taut band or a local twitch response may have further supported the identification of MTrPs; however, when surveying human literature, there was limited consensus on case definition of MTrPs apart from patient pain being recognized in the majority of studies (43).

This study provides initial evidence that between the two examiners in this study, and in this population of dogs, there was moderate to substantial inter-examiner reliability for identifying the presence or absence of MTrP in a hindlimb muscle group. However, defining the precise location of MTrPs remains challenging. The cranial thigh was found to most frequently contain MTrPs as well as the highest number of MTrPs of the hindlimb muscle groups palpated in this study. Given the growing recognition of myofascial pain as a potential contributor to chronic pain syndromes, especially in aging or mobility compromised dogs, refining diagnostic approaches and standardizing palpation techniques are crucial. Further research is needed to understand if these findings apply to a broader population of dogs and between more examiners of varying experience.

## Data Availability

The raw data supporting the conclusions of this article will be made available by the authors, without undue reservation.

## Supplementary material

Supplementary Video_1 Video demonstrating the jump sign, the primary criteria for identification of a myofascial trigger point.
